# Chromosome 1p and 11q Deletions and Outcome in Neuroblastoma—A Critical Review

**DOI:** 10.4137/cmo.s391

**Published:** 2008-05-20

**Authors:** Ricardo J. Komotar, Marc L. Otten, Robert M. Starke, Richard C. E. Anderson

**Affiliations:** Department of Neurosurgery, Columbia University, New York, NY

Neuroblastoma is an embryonic neoplasm of the sympathetic nervous system that accounts for approximately 15 percent of all pediatric cancer fatalities. Genomic change in neuroblastoma has been shown to correlate with both behavior and outcome. More specifically, amplification of the MYCN oncogene occurs in about 20% of neuroblastoma patients and has been shown to provide important prognostic information. This correlation has typically led to a multidisciplinary approach in the treatment of this condition, including preventive screening, dose-intensive chemotherapy, stem-cell transplantation, targeted therapies (radiation, monoclonal antibodies, prodifferentiation agents), and monitoring of minimal disease. However, although MYCN has been associated with high-risk tumors, more than 60% of patients with aggressive disease do not exhibit this mutation, suggesting that other genetic mechanisms are involved in the pathophysiology of neuroblastoma. Consequently, the development of additional genetic markers could be useful not only in further understanding the molecular biology underlying these tumors, but also defining more effective therapy and aiding prognosis in this patient population.

To this end, Attiyeh and colleagues published the results of their study investigating the effects of loss of heterozygosity (LOH) at chromosomes 1p and 11q on relapse and survival in neuroblastoma (1). LOH at chromosome arms 1p and 11q are noted frequently in neuroblastoma, and previous studies have suggested an association with high-risk features. Furthermore, 11q23 LOH is rarely observed in association with MYCN amplification. Given this fact, Attiyeh and colleagues hypothesized that 11q23 LOH could be a useful prognostic marker. In this study 915 samples of neuroblastoma were screened for LOH at chromosome bands 1p36 and 11q23, and associations with event-free and overall survival were performed.

Results were as follows: patients in whom tumors showed 1p36 LOH had three-year event-free and overall survival rates of 47% and 64% respectively, as compared with 77% and 85%, respectively, in those patients who did not have 1p36 LOH. Similarly, unbalanced 11q LOH (unb11q LOH) was strongly associated with both decreased event-free and decreased overall survival. Analysis of the subgroup of cases without MYCN amplification showed that both 1p36 LOH and unb11q LOH were highly associated with decreases in both event-free and overall survival. More specifically, patients with unb11q LOH had three-year event-free and overall survival rates of 50% and 66%, respectively, as compared with 74% (P < 0.001) and 83% (P < 0.001) in those that did. Finally, in a multivariate analysis, unb11q LOH was found to be independently associated with decreased event-free survival. Based on these findings, the authors conclude that 1p36 and unb11q LOH are risk factors that should be incorporated into clinical trials of neuroblastoma.

This study highlights the growing importance of genetic markers for use in stratification of tumor behavior. A number of markers including, MYCN amplification, HER2/*neu* over-expression, chromosome 17 gain, Trk expression, and CD44 genetic aberrations have proven useful, and the ability to detect risk factors at the time of diagnosis and tailor therapy accordingly could make the treatment of cancer both more effective and less toxic. Of greatest concern in neuroblastoma is the rare patient who despite a favorable clinical profile and no MYCN amplification progresses to high-risk disease. This study, although somewhat limited by the small numbers within subgroups, demonstrates that 1p36 LOH and unb11q LOH are strongly associated with outcome in patients with neuroblastoma. It is the hope of the authors’ that recognition and appreciation of these additional genetic markers will allow clinicians to more effectively tailor currently used prognostic schemes and treatment regimens.

## Figures and Tables

**Figure 1:**
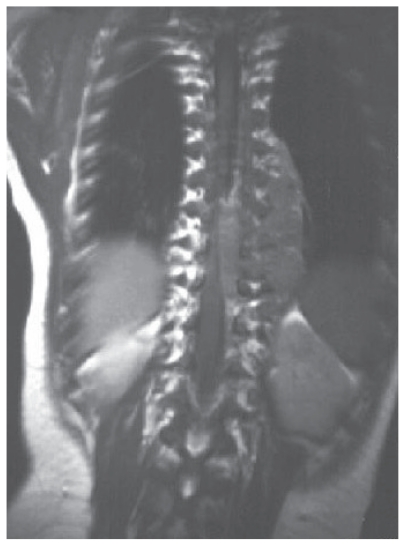
Coronal T1-weighted image demonstrating a large, left, paraspinal neuroblastoma in the mid/lower thoracic region.

## References

[1] Attiyeh EF, London WB, Mossé YP, Wang Q, Winter C, Khazi D, McGrady PW, Seeger RC, Look AT, Shimada H, Brodeur GM, Cohn SL, Matthay KK, Maris JM (2005). Children’s Oncology Group. Chromosome 1p and 11q deletions and outcome in neuroblastoma. N. Engl. J. Med..

